# The State of Digital Interventions for Demand Generation in Low- and Middle-Income Countries: Considerations, Emerging Approaches, and Research Gaps

**DOI:** 10.9745/GHSP-D-18-00165

**Published:** 2018-10-10

**Authors:** Dustin G. Gibson, Tigest Tamrat, Garrett Mehl

**Affiliations:** a Department of International Health, Johns Hopkins Bloomberg School of Public Health, Baltimore, MD, USA.; b Department of Reproductive Health and Research, World Health Organization, Geneva, Switzerland.

## Abstract

The recent introduction of digital health into generating demand for health commodities and services has provided practitioners with an expanded universe of potential tools to strengthen demand and ensure service delivery receipt. However, considerable gaps remain in our understanding of which interventions are effective, which characteristics mediate their benefit for different target populations and health domains, and what is necessary to ensure effective deployment. This paper first provides an overview of the types of digital health interventions for demand generation, including untargeted client communication, client-to-client communication, on-demand information services, personal health tracking, client financial transactions, and targeted client communication. It then provides a general overview of 118 studies published between January 1, 2010, and October 3, 2017, that used digital interventions to generate demand for health interventions. The majority (61%) of these studies used targeted client communication to provide health education or reminders to improve treatment adherence, and the most frequently (27%) studied health condition was HIV/AIDS. Intervention characteristics that have been found to have some effect on gains in demand generation include modality, directionality, tailoring, phrasing, and schedule. The paper also explores new emergent digital approaches that expand the potential effect of traditional demand generation in terms of personalization of content and services, continuity of care, and accountability tracking. Applying existing frameworks for monitoring and evaluation and reporting, research on emerging approaches will need to consider not only their feasibility but also their effectiveness in achieving demand generation outcomes. We propose a research agenda to help guide the field of digital demand generation studies and programs within a broader health systems strengthening agenda, including establishing and documenting the influence of intervention characteristics within different populations and health domains and examining the long-term effects and cost-effectiveness of digital demand generation interventions, as well as equity in access to such interventions.

## Abstract (precis)

Despite advances in digital technology to generate demand for health services, considerable gaps remain in our understanding of which interventions are effective, which characteristics mediate their benefit for different target populations and health domains, and what is necessary to ensure effective deployment. Future research should examine the long-term effects of, equity in access to, and cost-effectiveness and efficiency of digital demand generation interventions.

## INTRODUCTION

Global efforts to strengthen health systems have predominantly focused on ensuring adequate supply of, and access to, health commodities and services. Yet inadequate coverage on several key indicators of health use persists. Interventions that generate awareness and demand for health commodities and services in an equitable manner are often overlooked but show much promise for addressing ‘last mile’ populations and achieving the Sustainable Development Goals. Additionally, by increasing demand for health services, there is the potential to improve the quality of health care and to reduce inequities in health services.

The unprecedented reach and use of ubiquitous mobile technology by the general population^1^ has driven interest in leveraging digital health for demand generation. Its widespread use and growing sophistication of technical functionality has spawned an array of novel digital approaches to generate demand. Yet the health effects of these interventions have neither been documented nor reported at the same pace as implementation of the digital innovations. The substantial diversity of deployment and generalizability characteristics and factors associated across intervention study design and research settings may explain some of the observed heterogeneity in the reported effects. This diversity in implementation practices within limited settings not only has hindered comparisons across outcomes but has also resulted in a limited understanding of the conditions in which digital demand generation approaches can impact health outcomes.

This paper explores the variety of ways in which digital tools have been use to create demand in low- and middle- income countries (LMICs) as well as the factors that have contributed to variability in the evidence base. Additionally, we offer the reader guidance for documenting specific characteristics of demand generation interventions, an overview of emerging approaches aimed at strengthening the potential value of digital demand generation, and a proposed research agenda to further advance this promising field.

## TYPES OF DIGITAL HEALTH INTERVENTIONS FOR DEMAND GENERATION

Interventions that generate awareness and demand for health services are a staple in public health.^2^^–^^5^ Demand generation activities typically fall under 3 categories: (1) enlisting new users to adopt new health behaviors and services, (2) increasing demand among current users, and (3) taking market share from competing or inappropriate behaviors and products.^6^ The recently released World Health Organization (WHO) Classification of Digital Health Interventions^7^ categorizes the ways in which digital and mobile technologies can address health sector needs and strengthen both health systems and health outcomes. In the following section, we use this classification system to provide examples of digital demand generation interventions for untargeted client communication, client-to-client communication, on-demand information services, personal health tracking, client financial transactions, and targeted client communication.

### Untargeted Client Communication

Untargeted client communication, often referred to as mass communication, is defined here as the transmission of untargeted health promotion content in which relatively undifferentiated audiences receive identical communication material.^7^^,^^8^ Traditionally, untargeted client communication has been conducted through mass media techniques that can easily reach the general public, such as billboards, television, radio, and newspapers.^9^^,^^10^ The penetration of mobile technologies has since introduced new communication channels to this repertoire of mass communication strategies, particularly through the use of short message service (SMS)/text messages and social media platforms that are able to accommodate a large volume of users. This type of mass engagement has been used during disease outbreaks by ministries of health to transmit generic messages to notify constituents about a public health threat. For example, during the Ebola virus disease epidemic of 2014, the Ministry of Health and Social Action in Senegal transmitted 4 million SMS messages to the general public warning of the dangers of Ebola and telling them how to prevent contracting the disease.^11^ Similarly, health promotion campaigns have used untargeted client communication to notify the general population about available medical services or broadcast general information about health behaviors.^12^ Due to limited knowledge about the recipients, untargeted client communication usually contains generic content and, therefore, does not address the needs of specific demographic groups. As a result, few evaluations have explored the effectiveness of untargeted client communication using mobile technologies; much of the evidence on untargeted client communication is tied to traditional mass media methods.^9^^,^^10^^,^^13^^,^^14^ However, the pervasiveness of mobile technologies provides an opportunity to customize health promotion messaging, potentially shifting the emphasis away from mass communication channels to more targeted client engagement interventions.^9^

The Senegal MOHSA sent >4 million SMS messages during the 2014-2015 Ebola outbreak to warn the population about the dangers of the disease and provide prevention guidance.

### Client-to-Client Communication

Client-to-client communication, also known as peer communication, is the interaction between people who share common attributes, such as having similar health conditions, demographics, or prescribed treatments.^7^ By harnessing these empathetic and supportive interactions, client-to-client communication is uniquely positioned to influence behavior change and generate demand. Early forms of digital peer communication took place in online platforms such as social media networks, chat groups, and blog communities. For example, QuitNet—a smoking cessation program connecting current and former smokers—was one of first digital peer-communication mechanisms conducted over a web-based platform.^15^ While this virtual social support group model has been replicated across a variety of health areas, the general effectiveness of online groups on behavior change depends on a variety of factors, including a participant's level of engagement.^15^^–^^18^ Client-to-client communication conducted via mobile technologies, such as SMS and social media platforms, continues to grow in LMICs.^19^^–^^22^ For example, Project Khuluma, in South Africa, illustrates the use of mobile phones as the way a closed user-group—in this case, adolescents living with HIV—is able to communicate among themselves.^19^

### On-Demand Information Services

On-demand information services—defined here as health information accessible to the general public when triggered by the client^7^—are available via websites, helplines, SMS/text-messaging menus, or client applications, among other channels, and may be used to inform decision making. However, the defining feature of on-demand information services is that it relies on individuals to proactively initiate contact with the health system or information service. In its online form, on-demand information services can be as pervasive as the use of medical information websites such as WebMD. Increasingly, these types of health information services are being optimized for mobile devices and use in areas with low Internet connectivity, thus overcoming barriers related to Internet accessibility. Through these mobile phone-based modalities, clients can access health information by sending a message to an advertised number known as a short code. Documented examples of this intervention include platforms used by the Engage-TB approach and the Mobile for Reproductive Health (M4RH) project, whereby clients navigate through a menu of options by texting codes to specify the type of information they are seeking.^23^ The use of on-demand information services enables clients to determine the timing and content of the health information and reduces concerns related to client confidentiality, a common challenge associated with targeted client communication.^24^^–^^26^ Evidence for this type of client engagement in LMICs, particularly related to sexual and reproductive health,^27^ is emerging; studies to date have demonstrated its effectiveness in improving client's knowledge and awareness of health behaviors.

The use of on-demand information services enables clients to determine the timing and content of the health information and reduces concerns related to client confidentiality.

### Personal Health Tracking

Personal health tracking uses digital devices, such as mobile applications, wearables, and sensors, to document and monitor a client's health status.^7^ Clients may use personal health tracking services in a variety of ways, including accessing their own health records, monitoring their health or diagnostic data through wearable tracking tools, and actively documenting or journaling their health information. This approach to demand generation requires clients to proactively monitor their health data and facilitates increased personalization of their health care. However, more evidence on the effectiveness of this digital approach for improving health behaviors and linkages to health services is needed, and questions remain about the potential harm of unregulated digital applications for personal health tracking.^28^^,^^29^

### Client Financial Transactions

Client financial transactions are another common mechanism to generate demand for the use of health care services and to improve health outcomes. Traditionally, these type of transactions included the provision of vouchers, monetary incentives, or nonmonetary incentives to assist with out-of-pocket payments incurred by clients when seeking health services. With increasing mobile phone penetration, financial services—including savings, insurance, credit, banking, and payments—have rapidly been integrated into mobile phone applications and mobile network operators in what is known as mobile money.^30^ Although several systematic reviews have concluded that conditional and unconditional cash transfers and small incentives can improve health outcomes in LMICs,^31^^–^^34^ only a few studies have provided transfers via mobile money, airtime, or mobile-redeemable vouchers.^35^^–^^37^ Existing evidence suggests that the timing, frequency,^37^ and modality (i.e., airtime or mobile money)^38^ of mobile-money transfers are important factors that affect the efficacy of client financial transactions on health outcomes. Studies using conditional incentives have also found differences in health outcomes and health care use by the incentive amount, although these studies did not use mobile money.^39^^–^^42^ In one randomized controlled trial that provided conditional mobile-money incentives to Kenyan caregivers, the US$2.50 incentive was found to have significantly increased the proportion of children who were fully vaccinated, while the US$1.00 incentive that was given to a randomized group showed no significant effect.^43^

### Targeted Client Communication

Targeted client communication is defined here as the transmission of targeted health information “in which separate audience segments (often demographic categories) benefit from a shared message.”^7^^,^^8^ Targeted communication can also be further customized according to an individual's specific needs, resulting in “tailored client communication,” whereby message content is matched to the needs and preferences of an individual.^8^ While this type of communication can be unidirectional and bidirectional, initial contact is from the health system. This contrasts with on-demand information service to clients, where the client initiates the first contact with the health system. In high-income countries, targeted client communication sent via postal mail, automated telephone calls, email, and, more recently, SMS messages, have been successful at increasing several forms of health care use.^44^^–^^49^ For example, a systematic review found that reminder and recall interventions increased immunization rates by 5% to 20%.^45^

Targeted client communication originates from the health system and can be tailored to address the needs and preferences of an individual client.

Although postal and email reminders have demonstrated value in high-income countries, limited access to such services in LMICs hinders their implementation and viability. The increasing global availability of mobile phones provides alternative avenues for targeted client communication through the use of SMS and voice messages, mobile phone apps, and social media channels. Moreover, a growing body of literature has documented the efficacy of digital targeted client communication interventions, particularly through SMS, to improve health system performance and health outcomes in LMICs.^50^^–^^55^ MomConnect, a nationally scaled program supported by the South African Department of Health, provides a series of demand generation services to communicate stage-based pregnancy information to pregnant women and new mothers via SMS messaging and a mobile website. From August 2014 through April 2017, MomConnect registered 1,159,431 pregnancies, which corresponded to half of women attending their first antenatal care visit.^56^

## Overview of Digital Targeted Demand Generation Studies

We conducted a landscape literature review to identify published studies that used digital client communication to improve health outcomes in LMICs (Box). Since the seminal publication in 2010, which found SMS reminders improved antiretroviral drug adherence and lowered HIV viral loads in Kenyan adults,^57^ the number of published studies examining digital client communication has increased from 16 in 2011–2012 to over 50 studies in 2015–2016 (Table 1).

BOX **Literature Review Methods**

On October 3, 2017, we searched the PubMed database for the following terms:

- ("text message" OR "text messaging" OR "short message service" OR "SMS" OR "text reminder" OR "voice reminder" OR "voice message" OR "interactive voice response" OR "IVR" OR "mHealth") AND
- (“Randomized” OR “randomised” OR “RCT”) AND
- Country names for all LMICs

Titles and abstracts were screened for the following inclusion criteria:

- Randomized study design with a comparison group
- SMS or voice messages used to remind or inform clients about a health behavior
- Study conducted in an LMIC, as defined by the World Bank
- Study published from January 1, 2010, to October 3, 2017

Data for included studies were abstracted by one of the authors and were tabulated for each of the following variables:

- Year of publication
- Region: Latin America, Eastern Mediterranean region, sub-Saharan Africa, East Asia Pacific, Southeast Asia, South Asia
- Health outcome: Treatment adherence was defined as studies sending messages (reminders or information) to promote a health behavior that did not require participants to attend a health facility. Health care use was defined as a study that required participants to attend a clinic for a provided service.
- Type of health condition

Limitations: This was not an exhaustive literature review and its findings should be interpreted with caution. We only searched 1 database, did not include information from gray literature, and only included English language articles. The aim of this review was to provide a general overview of intervention studies using SMS or voice messages to generate demand for health interventions. WHO is currently developing guidelines on the comparative value of different digital health interventions based on a series of systematic reviews that are currently underway.

Abbreviations: IVR, interactive voice response; LMIC, low- and middle-income country; RCT, randomized controlled trial; SMS, short message service.

**TABLE 1.** Summary of SMS and Voice Reminder Studies That Included Comparison Groups and Were Conducted in Low- and Middle-Income Countries (N=118)

	No. (%)
**Year of publication**	
2010	1 (0.8)
2011–2012	16 (13.6)
2013–2014	23 (19.5)
2015–2016	53 (44.9)
2017 (through October 3)	25 (21.2)
**Region**	
Latin America	9 (7.6)
Eastern Mediterranean (Iran, n=14)	19 (16.1)
sub-Saharan Africa (Kenya, n=12)	43 (36.4)
East Asia/Pacific (China, n=25)	25 (21.2)
Southeast Asia (Malaysia, n=5)	8 (6.8)
South Asia (India, n=9)	14 (11.9)
**Health outcome (n=130)**^a^	
Treatment adherence	79 (60.8)
Health care seeking	51 (39.2)
**Types of conditions (n=124)**^b^	
Reproductive health	11 (8.9)
Child health	14 (11.3)
Acute illness or behavior	15 (12.1)
Lifestyle	15 (12.1)
HIV/AIDS	32 (25.8)
Cardiovascular	13 (10.5)
Diabetes	9 (7.3)
Screening visits	5 (4.0)
Other	10 (8.1)

a Some studies examined both health care seeking and adherence.

b Some studies examined multiple types of health conditions.

Of the 118 studies identified, the majority (61%) used targeted client communication to provide health education or reminders in order to improve treatment adherence and other health behaviors that do not require clinic visits. In terms of geographic scope, 51% (n=60) of studies were conducted in 4 countries: China (n=25), Iran (n=14), Kenya (n=12), and India (n=9). The most frequently studied health condition was HIV/AIDS (n=32, 26%). The majority of identified studies were 2-arm studies, where the intervention's effect on the study outcome was compared to a control or standard of care group. Within this field, trials have evaluated the effect of client communication interventions on, for example, HIV treatment adherence,^57^^–^^59^ prevention of mother-to-child HIV transmission,^60^^,^^61^ HIV testing,^62^^,^^63^ and voluntary medical male circumcision (VMMC).^64^^,^^65^

With the likely exception of SMS reminders for HIV-treatment adherence,^66^^,^^67^ the small number of studies, diversity in study populations, and heterogeneity in study designs and intervention characteristics not only have hindered comparisons across outcomes but have also resulted in a limited understanding of the conditions in which digital demand generation approaches can impact health outcomes. WHO is currently developing guidelines on the comparative value of different digital health interventions; a series of systematic reviews will be conducted on these topics in order to inform recommendations.^68^

## KEY INTERVENTION CHARACTERISTICS THAT MAY INFLUENCE CLIENT COMMUNICATION

Approaches to digital demand generation are distinguished by characteristics of the intervention. Key intervention characteristics of digital client communication that have been studied and found to have some effect on gains in demand generation include: (1) modality, (2) directionality, (3) tailoring, (4) phrasing, and (5) schedule (Table 2). In this section, we briefly discuss each of these characteristics and provide examples of studies that seek to establish the relative gains by varying the intervention characteristic.

**TABLE 2.** Intervention Characteristics of Targeted Client Communication

Deployment Characteristic	Definition
Modality	To which communication channel (voice, SMS/text, social media platform) were messages sent?
Directionality	Were messages one-way or two-way; provider to client and/or client to provider?
Tailoring	Were messages sent with information specific to the client, such as messages that include the client's name, the nearest or most appropriate clinic to receive services, or address a particular set of risk factors.
Phrasing	Were messages sent to inform or to motivate a client?
Schedule	When and how frequently were messages sent? For example, SMS reminders sent at 10am, 3 days and 1 day before child's vaccination date.

Key intervention characteristics of digital client communication that have been found to have some effect on gains in demand generation include modality, directionality, tailoring, phrasing, and schedule.

### Modality

The vast majority of client communication interventions rely on SMS or USSD (unstructured supplementary service data), though interactive voice response and communication via social media platforms are gaining prominence.^69^^–^^72^ SMS, compared to voice, has been more frequently deployed because of its ability to function in the absence of a stable network signal. Few studies have directly compared the performance between voice- and SMS/text-based messages. One study found that SMS and voice reminders performed similarly with regard to increasing attendance at pediatric HIV appointments in Cameroon.^73^ The increasing use of social media platforms, such as Facebook Messenger and WhatsApp, in LMICs provides a growing user base that supports not only SMS/text-based messaging but also more sophisticated communication content and interactivity, which warrants additional research.

### Directionality

Interactive, or two-way, messaging may be preferred by implementers, as it allows for an exchange of information between client and provider and also between clients. In South Africa, participants randomized to either one-way SMS or interactive SMS groups had similar reductions in systolic blood pressure, compared to the control group.^74^ A study including Ghanaian female students found that the interactive SMS group had higher gains in reproductive knowledge than the one-way group at 3 months and 15 months follow-up; the secondary outcomes of self-reported pregnancy and risky sexual behaviors by study arm had mixed results.^75^ The type of health outcome or behavior and its frequency, whether a one-off or repetitive behavior, like treatment adherence, are likely factors in determining if one-way or interactive client targeted messages are needed. It is important that issues such as the modality and client privacy are considered, particularly with the use of SMS messaging for sensitive health conditions.

### Tailoring

Tailoring is defined as “any of a number of methods for creating communications individualized for their receivers with the expectation that this individualization will lead to larger intended effects of these communications.”^8^ Tailoring can be as simple as providing a client's name or as sophisticated as leveraging a stored record detailing client characteristics in order to adapt and personalize demand generation content to a client's evolving needs over time. In a sample of Zambia U-Report SMS platform subscribers, no observed differences were made in both self-reported and clinic-verified VMMC between control, non-tailored, and tailored SMS groups, where the tailored group received messages targeting their intention level for VMMC.^65^ In contrast, a randomized controlled trial conducted in South Africa and Uganda found that non-tailored reminders significantly improved VMMC.^64^ Similar to the Zambia U-Report, a study in Iran showed no differences in hemoglobin A1c levels between control, non-tailored, and tailored SMS groups in participants with type 2 diabetes.^76^ Despite these representative examples, the use of tailoring merits further consideration and research, particularly since it can target key client characteristics. Although numerous studies have assumed the benefits of tailoring targeted client communication in the design of the intervention, very few studies have sought to assess the added gains in the personalization of client-targeted communication.

### Schedule

The scheduling of messages is an important characteristic of targeted client messaging. Scheduling refers to the time of day, frequency, and timing of messages sent in relation to an event or behavior, such as an SMS sent 3 days before an immunization appointment. In rural Kenya, investigators found that participants who received weekly SMS reminders were more likely to adhere to HIV treatment at 48 weeks than those who were not sent reminders or those who were randomized to receive daily SMS reminders.^62^ In South Africa, the scheduling and phrasing of messages—motivational vs. informational—resulted in different effects. Participants who received 10 motivational messages had significant gains in HIV testing compared to those who received 3 motivational messages, whereas no differences were identified between informational messages and their frequency.^62^ In randomized trials lacking control groups, no significant differences were seen in smoking cessation rates between those who received high-frequency or low-frequency SMS in China^77^ and mammogram screening rates between those who received a single SMS and those who received 2 messages in Lebanon.^78^ These results highlight that there may be a minimum threshold for the number of messages to produce an effect as well as a saturation threshold if too many messages are sent.

Research results have highlighted that there may be a minimum and maximum threshold for the number of messages to produce an effect.

### Phrasing

Like scheduling, the phrasing of the messages has been associated with the uptake of health services. In the same Kenyan study described earlier, no significant differences were shown between short and long messages—“This is your reminder.” versus “This is your reminder. Be strong and courageous, we care about you.”—on HIV treatment adherence measured at 48 weeks.^59^ In South Africa, participants who received 10 motivational messages were more likely to seek an HIV test than those who received 10 informational messages.^62^ The informational messages simply provided statistics about HIV testing, whereas the motivational messages indicated that HIV was not a death sentence, free drugs were available, and you could live a long life with HIV. Although it is not clear if the phrasing of messages has had an impact on health-seeking behaviors, it is likely that the phrasing is specific to study populations and health outcomes. Qualitative studies should be conducted with potential message recipients to optimize the content of messages, particularly for sensitive conditions such as HIV.^79^

### Summary

The observed heterogeneity in digital demand generation efficacy studies may be explained by both the content and the characteristics of the intervention. To that end, it is important that researchers and practitioners document the details of digital interventions so others can replicate the studies and so we can better understand what elements of digital demand generation approaches do and do not work. To that end, the WHO mHealth Technical Evidence Review Group has published an mHealth evidence reporting and assessment checklist to help improve the quality and reporting of digital interventions.^80^ Lastly, although intervention characteristics are described through the example of targeted client communication, these characteristics can also be applied to other forms of demand generation, such as untargeted client communication, client-to-client communication, and on-demand information services.

## EMERGING APPROACHES

Although they have not yet been captured in the scientific literature, a number of emerging approaches aim to strengthen the value of digital demand generation interventions and reflect the evolving sophistication of digital solutions globally and the growing familiarity of users with digital tools locally. These approaches include integrating demand generation with systems focused on unique identification and persistent health records, capturing information about receipt and effect, leveraging new messaging platform channels to reach additional users, and using artificial intelligence for enhanced personalization, wearables, and natural language chatbots.^81^

With the advent of social media platforms and the integration of digital demand generation approaches with identification systems and persistent records, including electronic medical record systems, digital demand generation approaches could be better positioned to also account for services received. Such systems could facilitate informing actual demand generation content and support measuring the effectiveness of specific digital demand generation interventions on expected and unintended outcomes, which, in turn, could inform personalization strategies. Furthermore, digital demand generation interventions that support client registration can link clients into national record systems and populate reporting indicators. The challenge of integrating these systems is ensuring that ethical data standards and client privacy concerns are appropriately addressed.

Integrating digital demand generation approaches with identification and record systems could help inform demand generation content and support measurement of expected and unintended outcomes.

The increasing familiarity with SMS and interactive voice response, access to mobile-money systems, and the availability and use of WhatsApp, Facebook, Weibo, and other social media platforms has facilitated narrowcasting messages to specific target audiences and personalizing autonomous interactive demand generation messaging through natural language processing and use of artificial intelligence in LMICs. Increasingly, digital demand generation approaches will be able to apply algorithms to identify clients in need of information or at risk of not adhering to their treatment protocols, who would, for example, benefit from a customized combination of financial incentives and personalized messaging delivered through specific channels with content designed for the intended beneficiary based on their personal motivations, health profile, and history of care.

## A PROPOSED RESEARCH AGENDA

The growing evidence base has begun to show that many digital demand generation approaches offer value; however, to what extent depends on the health program area, message content, and population and intervention characteristics. Future research will need to establish and document the influence of intervention characteristics, as highlighted above, within different populations and health domain areas. Rigorous studies that evaluate novel applications of digital demand generation approaches are still needed, as are studies that assess the additive benefits of each of the described intervention characteristics. This could be done through randomized controlled trials or, for scaled programs, implementation science or adaptive randomized studies. Although the majority of the identified literature has come from efficacy trials, many of which could be described as small pilots, digital interventions are gradually being brought to scale. To ensure rigorous evaluations of digital health interventions along each stage of its maturity, WHO recently released a monitoring and evaluation guide for digital interventions.^82^

To ensure rigorous evaluations of digital health interventions along each stage of its maturity, WHO recently released guidance for monitoring and assessing digital interventions.

Aside from conducting additional efficacy trials, larger questions must be asked about the generalizability and equity of previously conducted studies (Table 3). Much of the research shows short-term improvements associated with messaging and financial transactions; however, additional studies are needed that examine the long-term effects of these interventions. Additionally, many uncertainties remain, questioning whether employing these demand generation approaches exacerbates existing inequities. In many published reports, investigators provided a mobile phone^59^ or required mobile phone ownership for enrollment.^61^^,^^83^^,^^84^ The digital intervention may differentially benefit those who have access to mobile phones, technology literacy, or can afford the service. As the tested digital health intervention shifts from solely being a research concept to a routine tool for strengthening health services, the intervention needs to not only generate demand but also be measured against an accurate denominator of all eligible populations and reflect on any disparities in accessing the intervention. Future research should address sensitivities surrounding equity and potential disparities that may be perpetuated by digital demand generation approaches, including how device ownership or sharing may mediate the reach of the intervention. Lastly, additional studies that examine the cost-effectiveness and efficiency of digital interventions, compared to currently used systems, will provide vested stakeholders with the information needed to bring digital demand generation approaches to scale.

**TABLE 3.** Generalizability Considerations of Intervention Studies

Characteristic	Considerations
Recruitment site	Were participants recruited from a clinic, the general population, an opt-in from mobile network operator service, or a promotional advertisement?
Enrollment eligibility	Were inclusion/exclusion criteria generalizable to a larger population (e.g., mobile phone ownership was not required to participate in study)?
Equity	Were study findings equal across all subgroups? Did interventions reach marginalized populations? Were there trends in sharing mobile phones across some populations, which could have mediated effects?
Follow-up length	At what time point was the efficacy of an intervention assessed?
Target denominator	Was the denominator for the target population a known entity?

## CONCLUSION

The recent introduction of digital health into demand generation has provided practitioners with an expanded set of potential tools to strengthen demand and ensure service delivery receipt. Although this paper primarily discussed the predominant forms of digital demand generation interventions in the published literature, the field of digital health is rife with innovations and continues to evolve with the rapid sophistication of digital tools. As the capabilities of digital tools advance, a new wave of research will need to explore the emerging trends of digital technologies and how they may be harnessed to improve demand generation. Applying existing frameworks for monitoring and evaluation and reporting, research on emerging approaches will not only need to consider their feasibility but also their effectiveness on achieving demand generation outcomes.

## Notes

**Peer Reviewed**

**Funding:** This work was made possible by a grant from the Aetna Foundation, with support from the World Health Organization and The Johns Hopkins University Global mHealth Initiative.
